# Low serum ferritin and G6PD deficiency as potential predictors of anaemia in pregnant women visiting Prime Care Hospital Enugu Nigeria

**DOI:** 10.1186/s13104-017-3051-5

**Published:** 2017-12-08

**Authors:** Godwill Azeh Engwa, Marcellus Unaegbu, Marian N. Unachukwu, Mary-Gloria C. Njoku, Kingsley N. Agbafor, Wilfred Fon Mbacham, Anthony Okoh

**Affiliations:** 1 Biochemistry, Department of Chemical Sciences, Faculty of Natural and Applied Sciences, Godfrey Okoye University, P.M.B 01014, Thinkers Corner, Enugu, Nigeria; 2 Department of Microbiology, Faculty of Natural and Applied Sciences, Godfrey Okoye University, P.M.B 01014, Thinkers Corner, Enugu, Nigeria; 3 Department of Sociology/Psychology, Faculty of Social and Management Sciences, Godfrey Okoye University, P.M.B 01014, Thinkers Corner, Enugu, Nigeria; 4 Department of Biochemistry, Ebonyi State University, P.M.B. 053, Abakaliki, Nigeria; 5 Laboratory for Public Health Research Biotechnologies, The Biotechnology Centre, University of Yaounde I, BP 8094, Yaounde, Cameroon; 6 SAMRC Microbial Water Quality Monitoring Centre, Department of Biochemistry and Microbiology, University of Fort Hare, Alice, South Africa

**Keywords:** Anaemia, Malaria, Pregnancy, G6PD deficiency, Serum ferritin, Haemoglobin concentration

## Abstract

**Objectives:**

Though iron deficiency is known to be a major risk factor of anaemia, the association of G6PD deficiency and malaria with anaemia still remains unclear. Hence, a cross-sectional study involving 95 pregnant women visiting Prime Care Hospital in Trans-Ekulu region of Enugu Nigeria was conducted to determine possible predictors of anaemia in pregnancy.

**Results:**

The prevalence of anaemia, malaria and G6PD deficiency were 53.7, 12.6 and 60% respectively. Low serum ferritin (OR 5.500, CI 2.25–13.42, *p* < 0.05) and G6PD deficiency (OR 0.087, CI 0.03–0.23, *p* < 0.05) were associated with anaemia in pregnancy. On the other hand, malaria did not significantly associate (OR 1.184, CI 0.35–3.97, *p* = 0.964) with anaemia in pregnant women. These findings showed high prevalence of anaemia among pregnant women with low serum ferritin level and G6PD deficiency as high risk factors of anaemia.

## Introduction

Globally, anaemia affects about 2 billion people of which over 800 million are children and pregnant women particularly in Africa and Asia [1, 2]. Of the estimated 1 million yearly deaths in Africa and South-East Asia, anaemia is reported to account for about 75% [3]. Anaemia in pregnancy is even more prominent in developing countries with an estimated prevalence ranging from about 56 to 61% [4]. It is estimated to be responsible for about 20% of maternal death in sub-Sahara Africa [5].

Anaemia is characterized by low haemoglobin (Hb) concentration in blood below the normal level [6]. Iron is a major and essential component of Hb affix in the porphyrin ring mainly to bind oxygen molecules thereby facilitating its distribution in the body which is required for cellular respiration and other cell functions [7]. Iron is stored in the form of ferritin which is an intracellular protein containing about 4000–4500 iron atoms, thus serves as iron store in the body [8]. Low serum ferritin level in the body has been shown to be associated with iron deficiency and eventually anaemia [9]. Apart from iron level and serum ferritin in blood, glucose 6 phosphate dehydrogenase (G6PD) deficiency [10] and malaria [11] are other factors of concern. Deficiency of G6PD enzyme results in decrease level of reduced glutathione (GSH) thus making the red blood cells (RBCs) vulnerable to oxidative damage and eventually haemolysis or anaemia [12]. Malaria during pregnancy is among the most common complications of pregnancy and highly prevalent in sub-Sahara Africa [13]. In malaria-endemic areas, especially sub-Sahara Africa, about 25% of pregnant women are estimated to be infected with malaria parasite [14]. Though most malaria infections in pregnant women may be asymptomatic, the resultant anaemia may be fatal [15] constituting a major risk of anaemia in pregnancy.

Though serum ferritin and malaria are well documented as potential risk factors of anaemia in pregnancy, limited data is available on the contribution of G6PD deficiency with other predictors of anaemia in pregnancy.

## Main text

### Methods

#### Study area/participants

This pilot study was carried out among pregnant women attending anti-natal care (ANC) at Prime Care hospital, Trans-Ekulu, Enugu, Nigeria. Prime care hospital is located in Enugu East local government area of Enugu with coordinates: 6°27′9.60″N 7°30′37.20″E.

#### Study design and inclusion criteria

This cross sectional study involved pregnant women of all trimester visiting ANC from April to May, 2015 who had not received any therapy for anaemia or iron supplement, not severely ill, no complications and are willing to participate were considered for inclusion and consecutively recruited for the study.

#### Sample collection and analysis

Five millilitre of venous blood was collected into EDTA tubes to estimate the Hb level, pack cell volume (PCV), RBC count, and mean cell haemoglobin (MCV) using haematological analyser CELL DYN 1800, Abott Laboratories Diagnostic Division, USA. Thin and thick blood films were prepared, incubated with 10% Giemsa stain by Sigma-Aldrich Germany for 10 min then examined microscopically for the presence of malaria parasite using Model 3000FF microscope by Fisher Scientific, USA. Serum ferritin level was determined according to the method of Worwood and collaborators [16]. The methaemoglobin reduction test of Brewer et al. [17] was used for the determination of G6PD deficiency.

#### Data and statistical analysis

Anaemia in pregnancy was defined as Hb less than 11 g/dL and further classified as mild, moderate, and severe anaemia with Hb measurement between 10.0 and 10.9 g/dL, 7.0 and 9.9 g/dL and less than 7.0 g/dL respectively. For the MCV, anaemia was defined as < 80 fL/cell while no anaemia or normal ≥80 fL/cell. A PCV of less than 30% was considered as anaemia while a PCV greater than or equal to 30% was considered normal. RBC count of less than 3.2 million/mm^3^ was defined as anaemia and above 3.2 million/mm^3^ was considered normal. Anaemia as defined by serum ferritin was considered at less than 30 μg/L while above 30 μg/L was normal.

The data was analysed using statistical package for social sciences (SPSS) version 16 and presented in tables. Frequencies and proportions of categorical variables were compared with Chi square tests for association. Predictive factors of anaemia were considered as the independent variables and anaemia as the dependent variable to determine the correlation coefficient (*r*) and significance by Pearson correlation. Logistic regression was employed to determine the odd ratio (OR of the various predictive factors of anaemia. Confidence interval (CI) was taken at 95% and significant differences were considered at p ≤ 0.05.

### Results

#### Baseline characteristics of pregnant women

As shown in Table 1, 95 pregnant women enrolled in the study among which 37 (38.9%) were in their first trimester, 35 (36.8%) in their second trimester and 23 (24.2%) in their third trimester. Their ages ranged from 20 to 35 years with a mean of 27.33 ± 4.21 years while the most frequent age was 30 years. The Hb level of the women ranged from 5.5 to 13.5 g/dL with a mean of 9.9 ± 2.04 g/dL.

**Table 1:** Baseline characteristics of the study

Parameters	Age (years)	Hb (g/dL)	PCV (%)	RBC (million/mm^3^)	MCV (fL/cell)	Serum ferritin (μg/L)	G6PD (%)
Mean	27.33	9.99	30.48	3.47	89.67	30.45	57.22
Median	28	10.5	33	3.74	90	30	56
Mode	30	11.5	35	4	90	30	40
SD	4.21	2.04	5.21	0.77	1.32	1.14	1.33
Range	15	8	17.5	2.77	84	48	48
Minimum	20	5.5	19.5	2.03	46	10	39
Maximum	35	13.5	37	4.8	130	58	87

*SD* Standard deviation

#### Prevalence of anaemia, malaria and G6PD deficiency

Among the 95 pregnant women, 51 were anaemic with a prevalence of 53.7% of which 14 (14.7%) was mild, 30 (31.6%) moderate and 7 (7.4%) severe anaemia. Malaria had a prevalence of 12.6% (12/95) while the prevalence of G6PD deficiency was 60% (57/95).

#### Association of anaemia with diagnostic parameters

Anaemia was more prominent in pregnant women of the age group between 20 and 29 years than those between 30 and 39 years but there was no significant correlation between age group and anaemia (*r* 0.231, CI 0.12–0.28, *p* = 0.166). Anaemia in pregnancy was mostly present in the second trimester, followed by the first trimester, and least in third trimester. Anaemia was not associated with the trimester of pregnancy (*r* 0.184, CI 0.71–0.87, *p* = 0.789). Also, the MCV did not correlate with anaemia in pregnancy (*r* 0.182, 0.25–0.44, *p* = 0.370). On the other hand, PCV level was associated with anaemia (*r* 0.875, CI 0.00–0.03, *p* < 0.05) as most of the women with anaemia had a PCV level below 30%. Similarly, anaemia in pregnancy was associated with RBC count below 3.2 million/mm^3^ (*r* 0.679, CI 0.00–0.03, *p* < 0.05). Results are summarized in Table 2.

**Table 2:** Demographic characteristics and predictors of anaemia among pregnant women

Variables	Anaemia status					r (95% CI)	*p* value
	Absent (%)	Mild (%)	Moderate (%)	Severe (%)	Total		
Age range (years)							
20–29	25 (43.1)	10 (17.2)	21 (36.2)	2 (3.4)	58	0.231 (0.12–0.28)	0.166
30–39	19 (51.4)	4 (10.8)	9 (24.3)	5 (13.5)	37		
Trimester							
First	17 (45.9)	7 (18.9)	11 (29.7)	2 (5.4)	37	0.184 (0.71–0.87)	0.789
Second	14 (40.0)	5 (14.3)	12 (34.3)	4 (11.4)	35		
Third	13 (56.5)	2 (8.7)	7 (30.4)	1 (4.3)	23		
MCV (fL/cell)							
80	35 (46.1)	11 (14.5)	26 (34.2)	4 (5.3)	76	0.182 (0.25–0.44)	0.370
80	9 (47.4)	3 (15.8)	4 (21.1)	3 (15.8)	19		
PCV (%)							
30	44 (73.3)	12 (20.0)	4 (6.7)	0 (0.0)	60	0.875 (0.00–0.03)	0.000
30	0 (0.0)	2 (5.7)	26 (74.3)	7 (20.0)	35		
RBC (million/mm^3^)							
3.2	42 (70.0)	9 (15.0)	7 (11.7)	2 (3.3)	60	0.679 (0.00–0.03)	0.000
3.2	2 (5.7)	5 (14.3)	23 (65.7)	5 (14.3)	35		

*r* Pearson correlation coefficient; *CI* confidence interval

#### Association of anaemia with risk factors (ferritin, malaria and G6PD deficiency)

Low serum ferritin was associated with anaemia in pregnancy (OR 5.500, CI 2.25–13.42, *p* < 0.05) as anaemia was not usually present when the serum ferritin level was above 30 µg/L, while it was mostly present when serum ferritin level was below 30 µg/L. G6PD deficiency correlated with anaemia in pregnancy (OR 0.087, CI 0.03–0.23, *p* < 0.05). There was little or no association between malaria and anaemia in pregnant women (OR 1.184, CI 0.35–3.97, *p* = 0.964). The results are summarized in Table 3.

**Table 3:** Predictors of anaemia in pregnancy

Variables	Anaemia status					OR (95% CI)	*r* (95% CI%)	*p* value
	Absent (%)	Mild (%)	Moderate (%)	Severe (%)	Total			
Ferritin (μg/L)								
30	33 (64.7)	8 (15.7)	7 (13.7)	3 (5.9)	51	5.500 (2.25–13.42)	0.454 (0.00–0.03)	0.00
30	11 (25.0)	6 (13.6)	23 (52.3)	4 (9.1)	44			
G6PD								
Deficient	14 (24.6)	11 (19.3)	26 (45.6)	6 (10.5)	57	0.087 (0.03–0.23)	0.537 (0.00–0.03)	0.00
Non-Deficient	30 (78.9)	3 (7.9)	4 (10.5)	1 (2.6)	38			
Malaria								
Present	6 (50.0)	2 (16.7)	3 (25.0)	1 (8.3)	12	1.184 (0.35–3.97)	0.054 (0.93–1.00)	0.964
Absent	38 (45.8)	12 (14.5)	27 (32.5)	6 (7.2)	83			

*r* correlation coefficient, *CI* confidence interval, OR: odd ratio

### Discussion

Anaemia in pregnancy remains a problem of public health concern especially in developing countries where it can lead to so many adverse conditions or consequences which can affect the productivity and reproductive capacity of women and also lead to maternal death [18]. The prevalence of anaemia in pregnancy was 53.7% of which moderate anaemia was the most prevalent (31.6%) while severe anaemia was the least (7.4%). This results fall within the range of previous data reported in developing countries which showed the prevalence of anaemia in pregnancy to range from 35.0 to 75.0% [19]. Also, previous studies in Nigeria [20–22], Ghana [23] and South East Africa [24–26] have shown the prevalence of anaemia to be in a close range to that found in this study.

One of the main contributing risk factors of anaemia is iron deficiency which accounts for about 50% of all cases of anaemia [27]. Serum ferritin has been used as a measure for iron deficiency since it serves as iron store in the body [28]. Malaria in pregnancy has also been shown to be characterized by secondary anaemia which is at risk to mother and off-spring [29]. This is as a result of malaria parasite that destroys erythrocytes during cell division and merozoite release [30]. Another risk factor of anaemia is G6PD deficiency whereby a deficiency in this enzyme makes erythrocytes to be vulnerable to oxidative damage hence, liable to haemolysis and eventually anaemia [31]. The evaluation of these parameters for association with anaemia showed low serum ferritin (OR 5.5) and G6PD deficiency (OR 0.087) as high risk factor of anaemia. Thus, low serum ferritin or iron deficiency is found to be the most prevalent risk factor of anaemia. This has been confirmed in other studies which have shown iron deficiency to be responsible for anaemia [32] and iron supplementation as a protective measure [33, 34]. G6PD deficiency which had a prevalence of 60% was also shown to be associated with anaemia. This confirms previous studies which have earlier shown this relationship [35, 36]. However, malaria which had a prevalence of 12.6% showed little or no association (OR 1.184) with anaemia in pregnancy and thus not a potential risk factor. This is in contrast to the findings of a study by Matangila and collaborators which showed asymptomatic *Plasmodium falciparum* infection with a prevalence of about 30% to be associated with anaemia in pregnancy [37]. It is possible that seasonal variation may have accounted for this difference in malaria prevalence as this present study was carried out between April and May, a period whereby the rainy season was at an early stage in Nigeria compared to the study of Matangila and collaborators which was conducted from July to August deep into the rainy season where malaria transmission was at its peak accounting for the high prevalence and hence association with anaemia. The low prevalence of malaria observed in this study was hence a positive factor that minimized the extent of pregnancy associated anaemia as the presence of malaria is a known predictor of anaemia [38].

In conclusion, anaemia was found highly prevalent in pregnant women attending antenatal care at Prime Care Hospital in Trans-Ekulu region of Enugu Nigeria and was strongly associated with low serum ferritin level and G6PD deficiency as potential risk factors.

### Limitations

This study is a pilot study and was focused on one hospital, thus the small sample size may be limiting to generalize the findings to a large geographic area. Also, the study was carried out between April and May, a period during the rainy season where malaria transmission is not at its peak, thus low prevalence of malaria.

## Abbreviations

WHO: World Health Organisation
Hb: haemoglobin
G6PD: glucose 6 phosphate dehydrogenase
GSH: reduced glutathione
RBC: red blood cell
MCV: mean cell volume
PCV: pack cell volume
ANC: anti-natal care
OR: odd ratio
SPSS: statistical package for social sciences
CI: confidence interval
